# Effectiveness and Policy Determinants of Sugar-Sweetened Beverage Taxes

**DOI:** 10.1177/00220345211014463

**Published:** 2021-05-26

**Authors:** L.L. Hagenaars, P.P.T. Jeurissen, N.S. Klazinga, S. Listl, M. Jevdjevic

**Affiliations:** 1 Radboud University Medical Center, Radboud Institute for Health Sciences, IQ Healthcare, Nijmegen, the Netherlands; 2 Department of Social Medicine, Amsterdam UMC—University of Amsterdam, Amsterdam, the Netherlands; 3 Department of Dentistry—Quality and Safety of Oral Healthcare, Radboud University Medical Center, Radboud Institute for Health Sciences, Nijmegen, the Netherlands; 4 Department of Conservative Dentistry, Translational Health Economics Group, Heidelberg University, Heidelberg, Germany

**Keywords:** SSBs, obesity, fiscal policy, politics, health policy, nutrition policy

## Abstract

Sugar consumption is on the rise globally with detrimental (oral) health effects. There is ample evidence that sugar-sweetened beverage (SSB) taxes can efficiently reduce sugar consumption. However, evidence alone is seldom enough to implement a policy. In this article, we present a narrative synthesis of evidence, based on real-world SSB tax evaluations, and we combine this with lessons from policy development case studies. This article is structured according to the Health Policy Analysis Triangle, which identifies a policy’s content and process and important contextual factors. SSB tax policy content needs to be coupled to existing problems and public sentiment, which depend on more aspects than aspects related to (oral) health alone. Whether or not to include artificially sweetened beverages, therefore, is not solely a matter of showing the evidence of their oral health impact but also dependent on the stated aim of a tax and public sentiment toward tax policies in general. SSB taxes also need to be in line with existing tax and decision-making rules. Earmarking revenue for specific (health promotion) purposes may therefore be less straightforward as it might appear. The policy process of creating context-sensitive SSB tax policy content is not easy either. Advocacy coalitions need to be formed early in the process, and stamina, expertise, and flexibility are required to get a SSB tax adopted in a specific community. This requires a meticulously considered SSB tax structure implementation process. Oral health professionals who want to lead the way in advocating for SSB taxes should realize that evidence-based arguments on potential effectiveness alone will not be enough to realize change. The oral health community can learn important lessons from other “doctor-activists” such as pulmonologists, who have successfully advocated for higher tobacco taxes by being visible in the public debate with clear messaging and robust policy proposals.

## Sugar Consumption Is on the Rise

Per January 2021, sugar-sweetened beverage (SSB) taxes cover less than 10% of the global population (Appendix 1), despite ample evidence that they can effectively address the increasing burden of (dental) diseases related to sugar consumption. Caries is the most common noncommunicable disease (NCD) worldwide and substantiates a high disease and economic burden to individuals and society. Meier et al. (2017) estimated that excessive sugar consumption causes 26.3% of the burden of dental disease, including caries. Oral health experts, therefore, argue to restrict sugar consumption to a maximum of 3% of energy intake (Sheiham and James 2014; Moynihan 2016). Reducing sugar also benefits the fight against the obesity epidemic, which causes substantive health inequalities and fiscally unsustainable health care systems (Organisation for Economic Co-operation and Development [OECD] 2019). Worryingly, sugar consumption is on the rise, with global per capita consumption projected to increase from 22.7 kg in 2018 to 24.2 kg in 2028.

SSBs are widely recognized as a leading source of sugar consumption. SSBs are also linked to other NCDs because of their low nutritional value (Singh et al. 2015). The actual price of SSBs is a major determinant of consumption. Hence, it is worrying that SSBs have become more affordable over the past decades, most notably in high-income settings (Blecher et al. 2017; Ferretti and Mariani 2019).

This critical review presents a narrative review of the literature on 1) the effectiveness and 2) the policy determinants of policies that aim to reduce SSB consumption. We point out first that SSB taxes seem to be highly effective, although real-world effects are heterogeneous. We then explore important policy determinants to take up SSB taxes, since evidence alone is seldom enough for policies to be adopted. Meyer and Lee (2015) argue that oral health scientists must not only conduct rigorous science but also communicate their work to the appropriate bodies to improve policy outcomes. According to Meyer and Lee, this applies in particular to translating the evidence on dental caries and sugar consumption to policymakers.

We present SSB tax policy determinants discovered in a number of case studies from various contexts. The case studies identified together provide a rich variety of insights into the implementation of SSB taxes. These findings are presented using the Health Policy Triangle as an analytical frame (Fig.). The Health Policy Triangle is a highly simplified representation of reality that stipulates the interaction between a policy’s content, the context in which a policy originates, and the process of agenda setting up to implementation. Actors with varying interests influence these elements to promote their preferred outcome. The framework has been validated for tobacco, alcohol, and junk food taxes (Elliott et al. 2020), and its main advantage is that it helps to systematically explore the place of politics in health policy. Its descriptive purpose makes it useful to provide an accessible overview of such elements, which is especially suitable for a critical review. We conclude with a discussion of the potential role of the oral health community in promoting SSB tax policies.

## Existing Evidence on the Impact of SSB Taxes on Oral Health

Food labeling, marketing restrictions, and SSB taxes are commonly commended public policies to reduce sugar consumption. Simulation studies highlight the potential of front-of-package food labeling, whereby, for instance, a nutrient score or traffic light system warns consumers if foods contain excessive sugar (Jevdjevic et al. 2021). Other studies have proven the effectiveness of banning SSB purchasing at workplaces (Basu et al. 2020) or restricting the purchasing of SSBs within the Supplemental Nutrition Assistance Program (Choi et al. 2020).

Simulation studies especially demonstrate the potential oral health benefits of SSB taxes. Table 1 summarizes the findings of these studies. It shows that the cost-effectiveness of such taxes is potentially high because they affect all individuals in a jurisdiction with a tax while implementation costs are marginal. A study that ranked prevention policies in the Netherlands, for instance, found that “junk food taxes” could avert most disability-adjusted life years of all prevention policies that are not yet deployed in the Netherlands (van der Vliet et al. 2020).

**Table 1.** Results of Simulation Studies that Investigated the Costs and Oral Health Benefits of Sugar-Sweetened Beverage Taxation.

Study	Setting	SSB Tax Rate	Population	Time Horizon	Oral Health Benefits	Costs
Briggs et al. (2017)	United Kingdom	Tiered tax (high tax for drinks with >8 g sugar per 100 mL, moderate tax for 5–8 g, no tax for <5 g)	>4 y	Not applicable	Annual DMFT incidence reduced by 149,378 (95% UI, 45,231–262,013; incidence reduction of 2.4 per 1,000 person years) per year	Not applicable
Jevdjevic et al. (2019)	The Netherlands	20%	6–79 y	Lifetime	2.13 (95% UI, 2.12–2.13) caries-free tooth years per person; on population level, prevention of 1,030,163 (95% UI, 1,027,903–1,032,423) caries lesions	Treatment costs averted €159.01 (95% UI, 158.67–159.35) million; tax revenues €3.49 billion; administrative costs for taxation €37.3 million
Schwendicke et al. (2016)	Germany	20%	14–79 y	10 y	Net caries increment on population level 0.75 million	Treatment costs averted € 80 million; tax revenues €37.99 million
Sowa et al. (2019)	Australia	20%	≥18 y	10 y	0.21 DMFT averted per person; on population level, 3.89 million DMFT averted	Treatment costs averted A$666 million, A$35.61 saved per person

DMFT, decayed, missing, and filled permanent teeth; UI, uncertainty interval.

SSB taxes may seem effective in simulation studies, but several variables need to be considered to assess real-world effects. On the basis of the literature, we identify 5 main aspects to interpret the convincing but heterogenous real-world effects of SSB taxes. First, the extent to which the tax increase is reflected in actual consumer prices must be considered. A review confirms that SSB taxes are passed through to consumers almost entirely, which is good news for the effectiveness of the policy (Griffith et al. 2019).

Second, SSB taxes in general seem to have the desired effect of reducing SSB consumption. A meta-analysis of real-world SSB tax evaluations has shown that the equivalent of a 10% SSB tax was associated with an average decline in consumption of 10%, with substantial heterogeneity between jurisdictions (Table 2). The largest effect was found in Chile, where SSB consumption declined by 23.6%, if observed effects are scaled to the expected effect of a 10% tax. This compares to smaller US SSB taxes, where a drop in consumption of 2.3% is expected with such adjustment of observed effects. Results are even more heterogeneous if they are not adjusted for tax size. For instance, a purchasing drop of 22% was reported in Philadelphia (Seiler et al. 2021), whereas purchasing decreased by 6% in Mexico, Chile, and the state of Washington. It is not entirely understood why such diverse effects can be observed. The tax size obviously matters, but this does not explain why results are so heterogeneous when adjusted for tax size. Behavioral economics may offer some guidance. Chetty et al. (2009) have shown that consumers underreact to taxes that are not salient. In their field experiment in a grocery store, posting tax-inclusive price tags reduced demand by 8%. Increases in taxes included in posted prices also reduced alcohol consumption more than increases in taxes at the register. More details about consumer reactions in the short and long term can be found in Appendix 1.

**Table 2.** Change in Taxed Sugar-Sweetened Beverage (SSB) Consumption for the Equivalent of a 10% SSB Tax, Posttax versus Pretax Period, or Taxed Jurisdiction versus Control Jurisdiction.^[Table-fn table-fn2-00220345211014463]^

Study	Outcome Measure of Included Studies	Change in Consumption, %	95% CI, %
Berkeley, United States (*n* = 3)	Intake (2), sales (1)	−4.9	−7.4 to −2.3
France (*n* = 1)	Purchases	−15.7	−16.4 to −15.1
Chile (*n* = 2)	Purchases	−23.6	−49.3 to 15.0
Mexico (*n* = 4)	Sales (1), purchases (3)	−9.3	−10.4 to −8.2
Other United States combined (*n* = 6)	Intake (4), sales (2)	−2.3	−11.2 to 7.4
Catalonia, Spain (*n* = 1)	Sales	−13.7	−19.9 to −7.0
Total	Intake (6), sales (5), purchases (6)	−10.0	−14.7 to −5.0

Results drawn from the meta-analysis by Teng et al. (2019).

Third, SSB taxes have supply-side effects. Producers have been shown to alter their advertising strategy, and SSB taxes have led producers to introduce products outside the scope of SSB taxes by reducing sugar content, which can ultimately reduce sugar consumption generically. This happened in Portugal, where an SSB tax was introduced in 2017. Beverages with less than 80 g of sugar per liter were charged at €8.22 per 100 L and beverages above this threshold at €16.46 per 100 L. The market share of beverages containing less than 80 g/L increased by more than 60% in 2017 (Goiana-da-Silva et al. 2018). The UK government focused on producer effects with its “soft drinks industry levy.” One year after implementation in 2018, the volume of soft drinks purchased did not change, but the sugar amount in those drinks was 10% lower, compared to a counterfactual estimated from preannouncement trends (Pell et al. 2021).

Fourth, substitution effects to other unhealthy foods need to be considered. Finkelstein et al. (2013) investigated this with consumer panel data and found there does not appear to exist much substitution to other sugary foods like candy or cookies.

Fifth, cross-border shopping should be acknowledged. Opponents often question the effectiveness of SSB taxes by speculating that people will buy SSBs elsewhere. However, cross-border shopping also implies travel costs. These costs increase as other jurisdictions are further away or difficult to enter, so only in certain border areas will this be an issue. We expect marginal effects even in border areas, because SSB taxes only cause a marginal price increase. Local US SSB taxes, for instance, have tax rates of $0.01 to $0.02 per ounce ($0.34–$0.68 per liter). Only when SSBs are bought in bulk can it be economical for individuals living near the border to buy SSBs across the border, but SSB beverages concern bulky items that are difficult to carry around. Also, SSBs are mostly purchased as part of general groceries or on-the-go. This is an important reason why there is so much controversy around the unhealthy food environment near schools. A study on the Philadelphia case proves our point. Cawley et al. (2019) found that the tax did increase purchases outside the city, but it reduced the frequency of adults’ SSB consumption by 31% nonetheless.

In conclusion, there is ample evidence to hold the assumption that SSB taxes are effective. SSB taxes nevertheless generate resistance. Opponents claim that SSBs contribute only a small amount to the overall diet; therefore, the focus on SSBs is perceived as unjustified. Opponents stress that SSB tax attempts failed due to limited support and highlight economic effects, such as their regressive character and potential loss of jobs. Such arguments are fragmented and incomplete, however (Backholer and Martin 2017). The heterogeneity of real-world results nevertheless suggests that the effectiveness of SSB taxes depends on aspects such as the tax content, its context, the processes of agenda setting up to policy implementation, and the viewpoints of different stakeholders (the elements of the Health Policy Triangle).

## The SSB Tax Policy Content

The policy content of SSB taxes primarily refers to the included products and the tax structure. Hagenaars et al. (2017) conducted a comparative analysis of unhealthy food tax policies and found that SSBs were included more often than other unhealthy foods. For example, Denmark did adopt a saturated fat tax in 2012, but it was abolished a year later because it was difficult to administer the wide variety of food products with varying levels of saturated fats. SSBs can be demarcated much more easily. Fruit juices and milk-based sugary drinks are often included, but freshly squeezed juices and regular milks are often excluded. Artificially sweetened beverages are in some cases included. To an oral health professional, this makes sense as these beverages harm dental hard tissues (Korte et al. 2019), but including calorie-free beverages does not hold up when the objective is to reduce overweight. We reflect further on these demarcation decisions in the policy process section.

Tax revenue has frequently been earmarked for specific purposes, such as health promotion programs. This makes sense because public acceptability of SSB taxes is generally higher if revenue is used for such initiatives. For example, a representative poll in the Netherlands found that 55% supported a tax if revenue was used for health initiatives, compared to 42% if revenue was not earmarked (Eykelenboom et al. 2020). Revenue is not often officially earmarked, however, because fiscal rules can be a barrier, as shown in Israel and Mexico (Tamir et al. 2018; James et al. 2020).

Another important characteristic of the tax content is the tax structure. For example, a notable difference can be observed between cases in the European Union (EU) and the United States. In the United States, taxes only have 1 tariff, independent of sugar content. In the EU, taxes generally have a tiered design. This means that SSBs with more sugar are subjected to a higher tariff, to incentivize producers to reduce sugar content (Hagenaars et al. 2019).

## The SSB Tax Policy Context

Policy context refers to situational, structural, exogenous, and cultural factors (Buse et al. 2012). In the EU, SSB taxes were adopted by various political parties, including the Conservative Party in the United Kingdom, the center-progressive la Republique en Marche party in France, and a Socialist minority cabinet in Portugal (Hagenaars et al. 2019). In the United States, only Democrat-run local governments have implemented SSB taxes (Paarlberg et al. 2017). The policy appears to be a no-go for Republicans, although Mosier (2013) observed how moderate Republicans cooperated with Democrats on an SSB tax proposal in Kansas in 2010. It failed, because several other measures were under consideration to address budget shortfalls caused by the financial crisis. With a negative public discourse toward the tax that emphasized the impact on businesses and individual choice, oppositional forces were able to choose other policies instead. The Kansas example highlights the cliché that SSB taxes raise revenue.

Fiscal need is an important structural factor (Hagenaars et al. 2017). The surprising announcement by the French government of an SSB tax in 2011 is an interesting example. Three problems contributed to opening a policy window here. First, a conservative member of parliament reported the decreasing competitiveness of the French agricultural sector. The parliamentarian proposed reducing wage costs in agriculture and suggested to cover the deficit with an SSB tax, arguing these products were not typical French products. After some media attention, an SSB tax was announced, but it was coupled to 2 other problems: the reduction of public health insurance deficit, which could jeopardize France’s credit rating, and obesity prevention. Subsequently, the tax was amended in its design and public health rationale. A majority in parliament only favored the tax when revenues were used to relieve wage costs in agriculture. A compromise was reached in which the tax rate doubled, diet sodas were included, and revenues were split between agriculture and health care (Le Bodo et al. 2019).

Existing institutional arrangements regarding taxation and political decision making are important exogenous factors. In the United States, consumption taxes can be levied at the federal, local, and state levels, but SSB taxes are thus far solely implemented locally. Local SSB taxes affect state budgets because most states already charge a general sales tax, which enables state legislatures to preempt local SSB taxes. This poses a barrier to SSB taxes because industry lobbyism is more prominent at the state level. Arizona, California, and Michigan have already preempted local SSB taxes (Pomeranz and Pertschuck 2019).

In the EU, SSB taxes are adopted nationally. National governments need to acknowledge EU single-market policies, which have previously impeded the Danish fat tax and the Finnish tax on sweets and ice cream because of concerns that these policies might express illegal state aid. SSB taxes have not experienced such issues. Most EU countries know a tiered tax design with thresholds of 5 and/or 8 g of sugar per 100 mL, following the UK SSB tax.

Public sentiment is an important cultural factor. Berkeley, California, is a progressive city that adopted many health policy primers before it became the first US city to adopt an SSB tax in 2014. Residents are skeptical of corporate influence in their local policymaking process. This public sentiment resonated in the campaign message “Berkeley versus Big Soda.” The soda industry fueled the contra-corporate sentiment, which placed massive advertisements against the tax in the public transport system. Residents were offended by these actions. Something similar happened in Mexico. Advertisements were published that read “no to the Bloomberg tax [Bloomberg Philanthropies funded campaigns] . . . don’t let a gringo tell you what to drink.” People perceived this as xenophobic and out of step with regular Mexican discourse (Nestlé 2015).

Public sentiment can turn against SSB taxes when their shortcomings are overt in public debate. Arguments around the loss of jobs, the regressive character of SSB taxes, and personal responsibility can have a strong hold, but such arguments can be mitigated. Backholer and Martin (2017) provide a rebuttal and argue that the loss of jobs is minimal because a shift to nontaxed beverages can be observed, which may explain why no change in employment was noted in the case of the Mexican SSB tax. They argue that the regressive character is minimal—studies point at less than US$5 per year—while the health impact is progressive. Arguments around personal responsibility may be mitigated by pointing to the legal role of the beverage industry to maximize sales and profits.

The Philadelphia SSB tax serves as an example of shifting contentious debate away from paternalism toward discussion about how to finance kindergartens, a problem that was already high on the agenda (Purtle et al. 2018). SSB tax policy entrepreneurs explicitly chose not to focus on health, because “nobody in Philly cares about health,” as a participant mentioned in the study by Hagenaars et al. (2020). This compares to the Cook County government that unsuccessfully framed the tax as an attempt to mitigate obesity. High distrust in the county government fueled opposition along the rhetoric that the real goal was simply to impose another tax. The structure of the tax did not help either. It included diet sodas, which people perceived hypocritical given the stated aim of obesity reduction (Chriqui et al. 2020).

## The SSB Tax Policy Process

Policy processes are characterized by their unpredictable nature. Political scientists nevertheless agree that any policy process will run through the phases of (political) agenda setting, policy formulation, policy adoption, implementation, and evaluation. Hagenaars et al. (2020) investigated the SSB tax policy process in Berkeley, Cook County, and Philadelphia and drew 6 lessons (Table 3).

**Table 3.** Six Lessons Drawn from the Study by Hagenaars et al. (2020).^[Table-fn table-fn3-00220345211014463]^

Lessons		Examples
1	The policy had to be coupled to problems that were already on the agenda, which were not necessarily health related.	In Philadelphia, SSB tax revenue was earmarked for prekindergartens, since a lack of funding for prekindergartens was high on the agenda for years already.
2	Policy framing had to align prevailing political sentiment.	In Cook County, people were not necessarily trustful of the county government, which generated a skeptical view toward the tax’s stated goal of reducing childhood obesity.
3	The policy had to align existing tax rules and decision-making rules.	In Berkeley, California, state law would have necessitated a two-thirds majority if tax revenue was earmarked for specific causes. Advocates circumvented this institutional arrangement by suggesting to set up an advisory committee that would provide nonlegally binding advice to the city council on the purpose of revenue.
4	Political decisions and flexibility were required during policy formulation.	In Philadelphia, diet sodas were included after the then presidential candidate Bernie Sanders published a column in which he stressed the regressive income effects of the tax. This was mitigated by including diet sodas, which are consumed more by people with a higher income.
5	Building an advocacy coalition had to occur upfront in the policy process.	In Cook County, supportive health organizations only became aware of the tax attempt after its official announcement. Consequently, they had only 8 wk to work on the campaign prior to the vote, which proved too little to educate the public and county commissioners.
6	The advocacy coalition had to be locally grounded and able to influence media.	In Berkeley, the supportive advocacy coalition consisted of local community leaders who successfully activated their precincts and were able to generate positive media attention.

The policy process of sugar-sweetened beverage (SSB) taxes introduced in Berkeley and Philadelphia—where the tax is still in place at this writing—was compared to the policy process of the SSB tax introduced in Cook County—where the tax was abolished 2 months after its implementation.

Lessons 1 and 2 relate to agenda setting and highlight the importance of coupling the policy to high-agenda items and aligning it with political sentiment. Lessons 3 and 4 relate to policy formulation. Policy content had to be thought out meticulously to be in line with existing institutional arrangements. This requires expertise and flexibility among policymakers, to ensure the technical feasibility of the tax but also to ensure it can be altered when unexpected events change the scene.

It is important to acknowledge the difficulty of the SSB tax policy process. Lessons 5 and 6 of the study by Hagenaars et al. (2020) stipulate that a supportive advocacy coalition needs to be built upfront and that it needs to be locally grounded. James et al. (2020) confirm these lessons in their case study of the Mexican SSB tax, where a high level of organization, cooperation, planning, and effort was necessary. Falbe et al. (2020) draw similar lessons in their investigation of the Berkeley case, emphasizing the importance of thorough and timely communications with retailers and distributors for smooth implementation.

## Stakeholders

Stakeholder analysis is an approach for generating knowledge about individuals and organizations to understand their interests and influence in policy (Buse et al. 2012). The set of stakeholders involved in SSB tax policies is, in principle, relatively small. The soda industry is a powerful opponent; health nongovernmental organizations (NGOs) often support SSB taxes and eventually politicians enact a tax. Tax administrators, distributors, producers, and retailers of SSBs are involved in tax collection. More actors get involved when the policy is coupled to other problems. In Philadelphia, important supporters were civil society organizations advocating for investment in kindergartens and public infrastructure because the tax would benefit these causes (Hagenaars et al. 2020). In France, agriculture was involved since a parliamentarian majority was only reached when half of revenue would benefit agriculture (Le Bodo et al. 2019).

The position of budget officials should be considered. A main goal of budget officials is to deploy a simple tax system with the lowest transaction costs as possible. A pure theory on tax collection favors the central collection of a limited number of simple taxes (Tiebout 1956). But an SSB tax with tariff thresholds and earmarked revenue is less straightforward than a nonearmarked flat rate tax. Ministries of finance are skeptical of SSB taxes when there is no fiscal need, and they often oppose complex tax structures, as Tamir et al. (2018) discovered in Israel.

SSB tax policy analyses show how important it is to organize a strong advocacy coalition. This applies to supporters and opponents. For instance, the soda industry response to the French SSB tax proposal was disorganized, which Le Bodo et al. (2019) identified as an important policy enabler. In contrast, prior to the announcement of the Cook County SSB tax, no organized supportive coalition was constructed, whereas opposition was effective at organizing a repeal campaign (Chriqui et al. 2020).

Advocacy coalitions need to be locally grounded and able to influence media coverage. This can be difficult for the soda industry and for large philanthropical organizations that support SSB taxes (e.g., Bloomberg Philanthropies), because people mistrust outside actors when their role is overt (Eykelenboom et al. 2019). Media coverage can drive public acceptance of SSB taxes if media characterize sugar consumption as an industry-driven problem. This played a particular role in the policy process of the UK soft drinks industry levy (Buckton et al. 2018).

## The Role of the Oral Health Community: Ready to Take the Lead?

The need for a radically different preventive approach has been proposed to address the rise of sugar consumption and its dental sequelae (Watt et al. 2019). To our knowledge, however, oral health professionals to date have not had a decisive role in the policy process of any SSB tax. This does not mean they were not involved. In Berkeley, the California Dental Association funded a poll that gave policy advocates the confidence they could win the referendum (Hagenaars et al. 2020). The same organization filed a 2020 ballot initiative for a statewide tax together with the California Medical Association, in response to California’s moratorium on local SSB taxes (Falbe 2019). This initiative failed to reach the ballot (Ballotpedia 2020), but it highlights the oral health community can play an important role.

We argue that oral health professionals and the oral health profession have an important role to play as SSB tax policy catalysts and coalize with other stakeholders (outside the oral health field) who share common interests in reducing sugar intake and its sequelae. Oral health advocates may not always share identical interests with other stakeholders. For instance, obesity experts may propose excluding diet sodas for such a tax, whereas oral health professionals may advocate for their inclusion. It may also be in the interest of the oral health profession to allocate SSB tax revenues to oral health programs, as proposed by Bridge et al. (2020). We consider this sympathetic, but it is important to realize that budget officials will not automatically be in favor, since earmarking any tax revenue to specific causes typically complicates tax collection.

Coalition forming of oral health advocates with (other) SSB tax proponents is essential. Therefore, if the purpose is to improve (oral) health outcomes, precedence should be given to whatever helps reducing people’s sugar intake, irrespective of the extent to which accompanying revenues would be reinvested in oral health programs. Proactive leadership, coalizing, and engagement in SSB tax policy debates will create windows of opportunity for ending the global neglect of oral health.

Oral health professionals can learn from other health professionals who have successfully advocated for stronger prevention policies. Burki (2017) described how “doctor-activists” are in a unique position to advocate for issues like smoking, obesity prevention, and the social determinants of health, because they see the consequences of health threats daily, their voice is trusted, they understand the epidemiology, and they are used to communicating complex information to nonexperts. Pulmonologists are known for their antitobacco activism in particular. Therefore, most empirical evidence on how health professionals successfully advocated for public health issues focuses on tobacco control. Their strategy mostly revolved around being visible in the public debate with clear messaging and robust policy proposals. The robustness and messaging of tobacco control measures in South Africa, for instance, benefited from the coalitions that health professionals had formed with civil society activists, affected patient groups, researchers, government officials, and media (Elliott et al. 2020). This resembles the policy determinants of SSB taxes, as summarized in the Figure.

**Figure.**
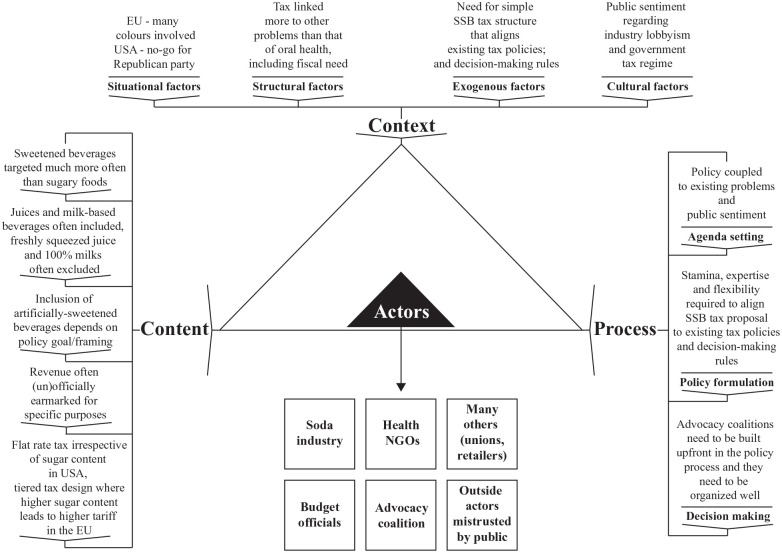
Policy determinants of sugar-sweetened beverage taxes, based on empirical analyses of real-world policy cases. Depicted using Buse et al.’s (2012) policy analysis triangle.

As a final point, the oral health community must also realize how they may be influenced by competing interests. Kearns and Bero (2019) highlighted conflicts of interest between dental research organizations and the sugary food and beverage industry, and they stated it is time for dental research organizations to develop and implement transparent, evidence-based policies and practices to eliminate or manage these conflicts of interest.

## Conclusion

The global rise of sugar consumption poses a threat to oral health and requires policy solutions. Ample evidence exists for SSB taxes to counteract this trend, but the oral health community should realize that other factors are at play than evidence-based arguments for oral health alone. This critical review has presented typical real-world patterns that can be observed in the policy content, context, process, and stakeholder behavior of SSB tax attempts. The oral health profession can and should play an important if not leading role in advocating for policies such as SSB taxes. They can, building on the findings on policy determinants for implementing SSB taxes presented in this review, follow suit to health professionals who were already able to act as successful prevention advocates.

## Author Contributions

L.L. Hagenaars, contributed to conception, design, data analysis, and interpretation, drafted the manuscript; P.P.T. Jeurissen, N.S. Klazinga, contributed to design, critically revised the manuscript; S. Listl, contributed to design, data analysis, and interpretation, critically revised the manuscript; M. Jevdjevic, contributed to conception and design, drafted the manuscript. All authors gave final approval and agree to be accountable for all aspects of the work.

## Supplemental Material

sj-pdf-1-jdr-10.1177_00220345211014463 – Supplemental material for Effectiveness and Policy Determinants of Sugar-Sweetened Beverage Taxes Click here for additional data file. Supplemental material, sj-pdf-1-jdr-10.1177_00220345211014463 for Effectiveness and Policy Determinants of Sugar-Sweetened Beverage Taxes by L.L. Hagenaars, P.P.T. Jeurissen, N.S. Klazinga, S. Listl and M. Jevdjevic in Journal of Dental Research
