# Supplementary material for: Effectiveness and Policy Determinants of Sugar-Sweetened Beverage Taxes
Source: J Dent Res. 2021 May 26;100(13):1444–51. doi: 10.1177/00220345211014463 (PMC8640336; doi:10.1177/00220345211014463)
Supplement: sj-pdf-1-jdr-10.1177_00220345211014463 – Supplemental material for Effectiveness and Policy Determinants of Sugar-Sweetened Beverage Taxes [file sj-pdf-1-jdr-10.1177_00220345211014463.pdf]

Appendix 1. Additional information

SSB taxes cover less than 10% of the global population:
As per January 2021, we could identify a tax to be in place in Brunei, Chile, Finland, France, Hungary, Ireland, Mexico, Norway, Peru, the Philippines, Portugal, Saudi Arabia, South Africa, Sri Lanka, Thailand, the United Arab Emirates, the United Kingdom and several countries in the Caribbean and Pacific. In the US, SSB taxes were implemented in Boulder, Philadelphia, Seattle and around the Bay Area. Note, however, that the list of jurisdictions with a tax is ever-changing.

SSB tax consumer reactions among different groups
Dubois et al. (2020) investigated SSB tax-induced behavior change while on-the-go in the UK. They found a larger effect among young people and people with lower incomes, but a smaller effect among those already consuming high amounts. Thus, SSB taxes seem more effective to prevent people from becoming high sugar consumers than they are to reduce the sugar intake of people with already high dietary sugar. Similar findings were reported for Catalonia, Chile and Philadelphia, with a more considerable decline in SSB purchasing among people with high income. This was not the case in Mexico, where households with lower incomes were most responsive (Griffith et al. 2019).

Long-term SSB tax consumer reactions
Long-term effects are less clear since most SSB taxes have only been implemented recently. Berkeley and Mexico are among the few geographies where longer-term trends have been investigated. In both cases a persistent drop in consumption was observed. Consumption declined by 0.55 SSBs per year in Berkeley after three years, compared to a pretax baseline of 1.25 (Lee et al., 2019). In Mexico, purchases of SSBs decreased by 5.5% in the first year and 9.7% in the second year (Colchero et al., 2017).
